# First Report of Paralytic Shellfish Toxins in Marine Invertebrates and Fish in Spain

**DOI:** 10.3390/toxins12110723

**Published:** 2020-11-19

**Authors:** Begoña Ben-Gigirey, Araceli E. Rossignoli, Pilar Riobó, Francisco Rodríguez

**Affiliations:** 1 Centro Oceanográfico de Vigo, Instituto Español de Oceanografía (IEO), 36390 Vigo, Spain; araceli.escudeiro.rossignoli@xunta.gal (A.E.R.); francisco.rodriguez@ieo.es (F.R.); 2 Centro de Investigacións Mariñas (CIMA), 36620 Vilanova de Arousa, Spain; 3 Instituto de Investigaciones Marinas, Consejo Superior de Investigaciones Científicas (IIM-CSIC), 36208 Vigo, Spain; pilarriobo@iim.csic.es

**Keywords:** *Alexandrium minutum*, invertebrates, HABs, paralytic shellfish toxins, PSTs, PSP, non-traditional vectors, Ría de Vigo, northwest Iberian Peninsula

## Abstract

A paralytic shellfish poisoning (PSP) episode developed in summer 2018 in the Rías Baixas (Galicia, NW Spain). The outbreak was associated with an unprecedentedly intense and long-lasting harmful algal bloom (HAB) (~one month) caused by the dinoflagellate *Alexandrium minutum*. Paralytic shellfish toxins (PSTs) were analyzed in extracts of 45 *A. minutum* strains isolated from the bloom by high-performance liquid chromatography with post-column oxidation and fluorescence detection (HPLC-PCOX-FLD). PSTs were also evaluated in tissues from marine fauna (invertebrates and fish) collected during the episode and in dolphin samples. The analysis of 45 *A. minutum* strains revealed a toxic profile including GTX1, GTX2, GTX3 and GTX4 toxins. With regard to the marine fauna samples, the highest PSTs levels were quantified in bivalve mollusks, but the toxins were also found in mullets, mackerels, starfish, squids and ascidians. This study reveals the potential accumulation of PSTs in marine invertebrates other than shellfish that could act as vectors in the trophic chain or pose a risk for human consumption. To our knowledge, this is the first time that PSTs are reported in ascidians and starfish from Spain. Moreover, it is the first time that evidence of PSTs in squids is described in Europe.

## 1. Introduction

Paralytic shellfish toxins (PSTs) are a group of natural neurotoxic alkaloids that are the causative agents of paralytic shellfish poisoning (PSP). These toxins are mainly produced by toxic dinoflagellates belonging to the genera *Alexandrium*, *Gymnodinium* and *Pyrodinium* and they have also been identified in some cyanobacteria which may occur in fresh and brackish waters [1]. PSTs act by blocking voltage-gated sodium channels, thus slowing or abolishing the propagation of the action potential, and, as a consequence, a progressive loss of neuromuscular function ensues. This leads to neurological symptoms: extremities numbness, tickling sensation in lips, tongue and mouth, breathing difficulty, gastrointestinal problems and a sense of dissociation followed by complete paralysis that could result in respiratory arrest and cardiovascular shock, or death [2,3].

PSTs are transferred and bioaccumulate throughout aquatic food webs, and thus can be vectored to terrestrial biota, including humans [4,5,6]. Fisheries closures and human intoxications due to PSTs have been documented not only in filter feeders (i.e., bivalve mollusks) but also in several non-traditional vectors. These include, but are not limited to, marine gastropods (both carnivorous and grazing), crustaceans (such as lobster and crabs) and fish that acquire the toxins through trophic transfer [7]. Although most filter feeders are relatively insensitive to PSTs [8], ingestion of contaminated shellfish can cause paralytic shellfish poisoning (PSP) syndrome. Therefore, it is essential to conduct regular monitoring of phytoplankton and shellfish tissues toxins content in shellfish harvesting areas. Such monitoring programs rely on intensive sampling and analysis that require the availability of rapid, sensitive, accurate and precise testing methods [9]. Although most monitoring programs focus mainly on bivalve mollusks, some countries have regulated PSTs levels in other marine vectors. This is the case of the EU where Regulation (EC) No 853/2004 sets the maximum PSTs concentrations in bivalve mollusks, echinoderms, tunicates and marine gastropods [10]. In recent years, several papers reported, for the first time, the presence of PSTs in non-traditional vectors in different European countries [11,12,13]. However, to our knowledge, studies describing the presence of PSTs on several non-bivalve mollusk marine invertebrate vectors have not been conducted in Spain.

**Figure 1:**
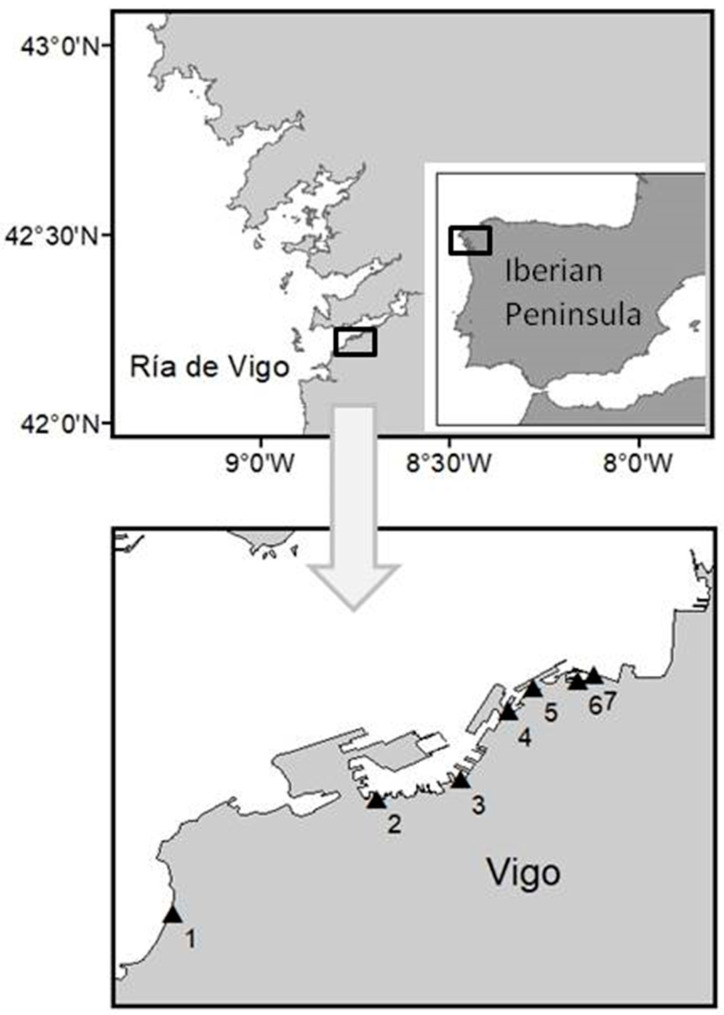
Upper panel: area of study and location of Ría de Vigo. Lower panel: sampling stations for seawater samples from the bloom (triangles) in the Vigo marina and Samil Beach. This Figure was processed with ArcGIS program version 10.5.1.7333, copyright 1999–2017 Esri Inc. (website http://www.esri.com).

The present study addresses an exceptional PSP outbreak in the Rías Baixas (Ría de Vigo and Ría de Pontevedra, both southern Galician Rías), northwest Spain (Figure 1), in summer 2018 caused by an intense and prolonged (~one month) *Alexandrium minutum* bloom. PSTs were analyzed in marine fauna (invertebrates, fish and dolphins), collected during the bloom in several of Vigo’s sampling points (Table 1). Analyses were performed by high-performance liquid chromatography with post-column oxidation and fluorescence detection (HPLC-PCOX-FLD). PSTs were quantified not only in bivalve mollusks, but also in mullets, mackerels, starfish, ascidians and squids.

The production of PSTs in the *A. minutum* strains isolated from the bloom was also evaluated by HPLC-PCOX-FLD. There were differences in the number of gonyautoxins (GTXs) (GTX1, GTX2, GTX3, GTX4) produced by the isolated strains and in the relative abundance of these compounds.

The results obtained deserve further attention towards the potential accumulation of PSTs in marine invertebrates other than shellfish and in fish that could act as vectors in the trophic chain or pose a risk for human consumption. They also stress the importance of intensifying the monitoring of marine invertebrates other than bivalve mollusks.

## 2. Results

### 2.1. Analysis of PSTs in Cultures from *A. minutum* Strains by HPLC-PCOX-FLD

**Figure 2:**
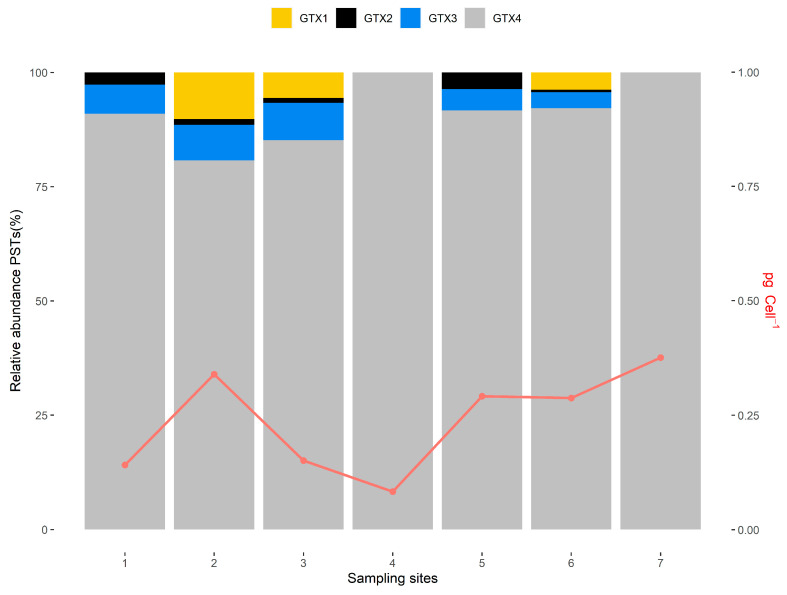
Relative abundance of the different paralytic shellfish toxins (PSTs) in each of the 7 sampling sites (as shown in Table 1), calculated as mean values of the several *A. minutum* strains isolated from each site. The red line indicates mean total toxicity levels per cell for each strain group.

Table 1 summarizes the PST results on 45 *A. minutum* strains isolated from the bloom. The analysis of these strains revealed a toxic profile including GTX1, GTX2, GTX3 and GTX4 toxins. The dominant toxin was GTX4: 34 out of 45 strains produced GTX4 levels ranging from 0.05 to 0.42 pg cel^−1^ (mean value 0.22 ± 0.11 pg cel^−1^). It is important to remark that GTX4 shows a relatively high toxicity equivalency factor (TEF), 0.7, according to the European Food Safety Authority EFSA [14]. Twenty-four strains produced GTX3, in the range of 0.001 to 0.08 pg cel^−1^ (mean value 0.02 ± 0.02 pg cel^−1^). Then, 10 strains produced GTX2, with levels ranging from 0.01 to 0.03 pg cel^−1^ (mean value 0.01 ± 0.01 pg cel^−1^). Finally, four strains were able to produce GTX1, with levels between 0.10 and 0.18 pg cel^−1^ (mean value 0.14 ± 0.04 pg cel^−1^). Only 4 strains were able to produce the four toxins (Table 1), while 11 strains did not produce any PSTs. Figure 2 shows the relative abundance of GTX1, GTX2, GTX3 and GTX4 in each of the seven sampling sites from where the strains were isolated (Table 1).

### 2.2. Analysis of PSTs in Fish, Invertebrates and Dolphins by HPLC-PCOX-FLD

A total of 35 samples were prepared for analysis. Table 2 summarizes the results from the samples that tested positive for PSTs. Six dead mullets were sampled in the bloom area. Two of them (mullet 1 and 2) were smaller and so deteriorated that separated analyses of muscle and viscera were not possible. For mullets 3, 4, 5 and 6, a separate analysis of the muscle and digestive tract was conducted. GTX3 was quantified in the digestive tract of mullets 3 and 6. However PSTs were not detected in the corresponding muscle samples. Two mackerels (immediately caught by fishermen) were obtained from the contaminated area and separate analyses were conducted for the muscle and digestive tract in both of them. PSTs were only detected and quantified in mackerel 2 (Table 2), with results showing higher levels in the digestive tract. Five fresh squid samples were analyzed and only one of them showed low GTX3 levels. Surprisingly, two starfish, separately analyzed, presented low GTX2 and GTX3 concentrations. All these samples were well below the legal limit regulated in the EU legislation for PSTs (800 µg equivalents STX·diHCl/kilogram) [10]. PSTs were not detected in any of the six dolphin samples tested (either stomach or stomach contents) or in the homogenate of a triggerfish found dead. It is worth mentioning the results from one ascidians sample, showing levels around half the EU legal limit for PSTs and the same toxic profile (GTX1, 2, 3 and 4) as the cultures established from cells isolated from the bloom.

The five bivalve mollusks samples evaluated had the highest PSTs levels (Table 2). With the exception of one oyster sample, all had toxicity levels above the maximum total toxicity PSTs levels regulated in the EU. Mussels and scallops samples showed the presence of the four GTXs together with NEO and/or STX. STX and NEO were not found in the analysis of the *A. minutum* strains isolated in the bloom. Recent PSP episodes in Ría de Vigo (between 2015 and June 2018) had only been detected in December 2016 and February 2017. They were linked to the presence of *Gymnodinum catenatum* according to the Galician monitoring program: Instituto Tecnolóxico para o Control do Medio Mariño de Galicia (INTECMAR, http://www.intecmar.gal; Xunta de Galicia). In Galicia, STX was only detected in certain samples at trace levels and NEO was not detected in bivalve mollusks contaminated during *G. catenatum* blooms when analyzed by the AOAC 2005.06 method [15]. For all these reasons, we do not expect that STX and/or NEO were accumulated in the shellfish prior to this bloom.

## 3. Discussion

### 3.1. PSTs in Cultures from *Alexandrium minutum* Strains by HPLC-PCOX-FLD

Differences in toxin profiles have been observed among the species belonging to the genus *Alexandrium* [16]. However, general characteristics can usually serve to identify the distinction from the toxin composition of other dinoflagellate genera (*Pyrodinium* and *Gymnodinium*). For instance, decarbamoyl PSTs (dcSTX, dcNEO, dcGTX1-4) and the N-21 sulfocarbamoyl analogues C3 and C4 are rarely found in *Alexandrium* species [17]. Within the *Alexandrium* genera, some species-specific toxin markers were identified for the *A. minutum* group [17]. This refers to the tendency to primarily or exclusively produce gonyautoxins (GTX1, GTX2, GTX3, GTX4). With regard to this marker, most of the clonal *A. minutum* strains isolated in this study showed the production of one or more of the GTXs toxins (Table 1). No other PSTs were generated by any of the strains.

Differences in toxin profiles have been found among strains of the same species collected from different geographical locations. Toxin profiles are influenced by physiological conditions such as nutritional conditions and the cell cycle stage, among others [16]. However, the production of a certain suite of toxins seems to be fixed genetically for each clonal strain of *Alexandrium*. In this study, we found differences in the number of GTXs derivatives present when analyzing the isolated strains (Table 1) and in the relative abundance of these compounds (Figure 2). In some strains, only GTX4 was present, while in others, two, three or the four GTXs derivatives were present. However, for certain strains, the toxins levels were close to the method detection limit. In a study that compared the toxin composition of *Alexandrium minutum* isolates from different geographical locations [18], the authors reported that the most common or geographically widespread toxin composition seems to be GTX1–4. They also reported the production of GTX1–4 toxins by Spanish isolates from Galicia and Mallorca. However, isolates from New Zealand, Denmark, the UK and Italy were also able to produce other PSTs such as NEO, STX or C toxins. A recent paper provides newer details on the toxins profiles associated with *A. minutum* species globally [19]. This study concludes that the most common toxins produced by *A. minutum* are GTX1 and GTX4, with GTX4 being the most dominant toxin in a range of strains which are widely dispersed geographically. Analysis conducted by K-means clustering on toxin profile data of strains from different geographical locations generated five clusters of PST toxin profiles [19]. The first cluster, in which Spain was included, is the most widely dispersed and predominated by GTX1 and GTX4. The strains analyzed in our study can be grouped, both geographically and in terms of the toxins detected, in cluster one with GTX4 as the predominant toxin. 

### 3.2. PSTs in Fish, Invertebrates and Dolphins by HPLC-PCOX-FLD

It is well known that PSTs are found not only in dinoflagellates and bivalve mollusk filter feeders, but can also accumulate in phylogenetically diverse groups of animals. For instance, they were reported multiple times in crabs, lobsters and marine snails [7,8,20]. PSTs can also cause mortalities in fish (mackerel, herrings, etc.), seabirds and marine mammals (humpback whales, monk seals, etc.) as they move through the marine food chain [8,21,22,23,24]. In the present study, we found different PSTs in mullets and mackerels samples up to a total toxicity of 292 µg equivalents STX·diHCl/kilogram. In general, PSTs are present in higher concentrations in the digestive tract of vector species (shellfish, crabs, snails and fish) than in their muscles [7,8]. Since fish are generally ingested without the digestive tract, and also for the fact that they can die before they reach dangerous levels in their flesh [8], the risk of PSP intoxication from fish seems lower. The three dolphins were brought to our laboratory because several dolphins were found stranded, and some died, during the summer of 2018. This prompted us to investigate if these events could have been related to the *A. minutum* bloom. However, we did not detect PSTs in any of the dolphin samples analyzed that could substantiate such correlation.

Two starfish samples and one squid sample presented low PSTs levels. One ascidian sample showed levels around half the EU legal limit for PSTs [10]. PSTs have been previously reported in other starfish (*Asterias amurensis, Asterina pectinifera*) and ascidians (*Holocynthia roretzi*) from Japan [8,25,26]. In the United Kingdom, the possible link between stranded starfish containing high PSTs levels with cases of PSP intoxication in dogs was suggested [27]. Other authors [12] published the presence of PSTs in several starfish samples collected during 2012–2013 in Madeira and Azores islands. They suggested these non-traditional invertebrate vectors could play a role in PSP episodes. In 2014, another study [11] described that six people were poisoned following consumption of fresh ascidians (*Microcosmus vulgaris*) harvested in the Adriatic Sea. However, to the best of our knowledge, there are no previous reports of ascidians, starfish or squids contaminated with PSTs in Spain. None of the recent articles [11,12,13] about PSTs in non-traditional vectors from European waters report the presence of PSTs in fish. Moreover, we could only find a Canadian paper reporting the presence of PSTs in the Humboldt squid (*Dosidicus gigas*) [28]. Therefore, the present contribution is, to our knowledge, the first report of PSTs in squids in Europe. Although PSTs accumulate mainly in the squid’s viscera [29], there are Mediterranean countries where the consumption of non-eviscerated small squids is common. Thus, some risk of intoxication could occur if PSTs concentrations are high. The results from our study reinforce the need to further evaluate the possible presence of PSTs in non-bivalve mollusk species, such as cephalopods, echinoderms and tunicates. Other researchers [7,12,30] have highlighted the importance of reviewing monitoring strategies for these groups in view of the increased interest in the exploitation of these marine live resources. Consumption of squids, barnacles, crabs, etc., is high in certain European areas and it is extremely important to prevent human intoxication caused by PSTs present in these vectors. This could be especially needed for cephalopods in some Mediterranean countries. Moreover, due to the fact that some ascidian species are considered a delicacy in North America, Japan, Korea, Chile, Peru, New Zealand, Spain, Italy and France and that human intoxications related to ascidians were already reported in Croatia [11], it seems crucial to examine their potential role as PSTs vectors. This is not the first time that PSTs are reported in marine invertebrates in Galicia. For instance, in 2012, a report [15] described the presence of PSTs (levels around half the EU legal limit) in the crustacean *Pollicipes pollicipes* collected during the 2005 *Gymnodinium catenatum* intense bloom. However, more data from research studies and monitoring programs are needed to evaluate the potential risks these species could pose for human’s health as well as their impacts in the trophic chain. They are extremely important in terms of risk assessment evaluation. These data are also essential since monitoring of PSTs in certain European and third countries is still mainly focused on bivalve mollusks, when scientific evidence suggests that it is needed to cover several marine invertebrate species too.

Our study findings come from an opportunistic sampling that took place while collecting *Alexandrium minutum* samples during the intense bloom. Every marine invertebrate and fish sample obtained at that time was analyzed and included in the present work. Unfortunately, monitoring vector species over time was not considered by then. This report provides a snapshot of the PSTs levels found in those organisms during the *A. minutum* bloom, calling the attention towards their accumulation in the trophic chain and the potential risks for human consumption. The present study was the trigger for our group to consider the need of a future exhaustive study involving several species and areas in our region.

## 4. Materials and Methods

### 4.1. Marine Fauna Sampling

**Figure 3:**
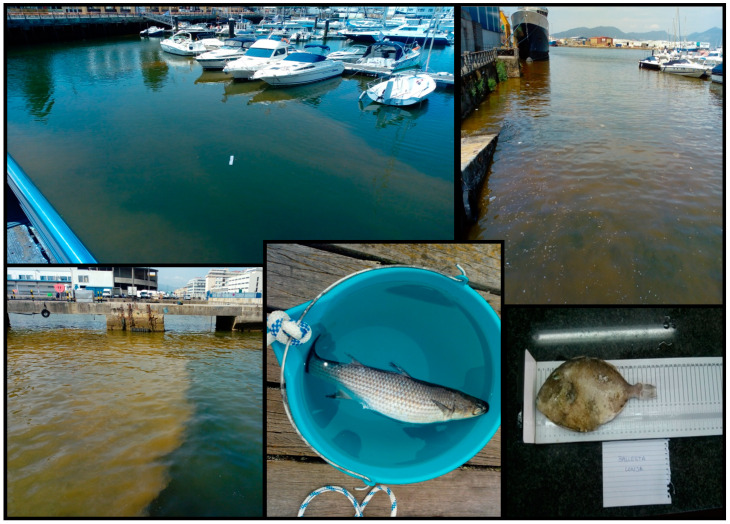
*Alexandrium minutum* bloom at different docks in Vigo, together with a mullet (**middle**) and a triggerfish (**right**) found dead during the toxic episode (photos by Francisco Rodríguez).

Invertebrates (starfish, ascidians, squids and bivalve mollusks) and mackerels were collected in sampling sites at the Vigo marina by a scuba diver (6: Náutico, Table 1) and local fishermen (3: Beiramar; Table 1). In addition, dead fish (mullets and a triggerfish (*Balistes carolinensis*)) observed during the bloom were collected with a bucket and by local fishermen (3: Beiramar); Table 1, Figure 3). Samples of three dolphins (*Delphinus delphis*) found dead in July and August 2018 were provided by the CEMMA (“Coordinadora para o Estudo dos Mamíferos Mariños”, http://www.cemma.org) and the Marine Mammals Department (IEO Vigo). Dolphins with codes DDE22, DDE 24 and DDE31 were found stranded in Cies Islands, Oia and Cangas. Cies Islands and Cangas are located in Ría de Vigo, and Oia is situated a little bit southward. Samples from the stomach and stomach contents were taken for analyses.

### 4.2. Isolation of *A. minutum* Strains

An extensive reddish color bloom was reported by first aid and rescue services at Samil Beach (Ría de Vigo, NW Iberian Peninsula) on the morning of 28th June 2018. Microscopical observations of seawater samples taken near the shoreline, using an Apstein-like net (20 µm mesh size), revealed a nearly monospecific bloom of *A. minutum* by means of epifluorescence microscopy and calcofluor staining [31] [Rodríguez et al. in prep.]. During subsequent dates up to the end of July, red waters were observed along the Vigo marina (Figure 3). Surface seawater samples for the isolation of A. *minutum* strains were collected with a bucket in the bloom area at seven sampling sites (Table 1).

Single A. *minutum* cells were isolated using a glass capillary pipette and poured into 96-microwell plates in L1 medium [32] without silicates, made with seawater from Ría de Vigo and salinity adjusted to 32. Plates were incubated at 19 °C with a photon irradiance of about 120 µmol·m^−2^·s^−1^ of PAR (LED illumination), measured with a QSL-100 irradiometer (Biospherical Instruments Inc., San Diego, CA, USA) and at a 12:12 L/D photoperiod. The obtained clonal cultures (Table 1) were maintained in 50 mL Erlenmeyer flasks for further characterization.

### 4.3. PSTs Analyses (*A. minutum* Strains and Marine Fauna)

Fish (mullets, mackerel and a triggerfish), starfish, squids, ascidians and dolphins were dissected in the laboratory and samples from different tissues (Table 2) were homogenized using an Ultraturrax (IKA) at 11,000 min^−1^. Bivalve mollusk (mussels, scallops and oysters) homogenates were prepared according to [33].

Sample extraction and deproteination were carried out according to [34]. When marine fauna samples were analyzed, interferences, due to naturally fluorescent compounds, were noted in some samples. The procedure used at the laboratory to check for naturally fluorescent peaks consisted of running the samples without the oxidation step. For this purpose, the oxidant and acid reagents were replaced by ultrapure water. In order to remove the interferences, a clean-up procedure was implemented. Solid-phase extraction cartridges (Sep-PAK^®^ Vac, 3cc, 500 mg tC18, Waters WAT036815) were conditioned with 6 mL MeOH, followed by 6 mL of deionized water. Then, 1 mL sample extract was loaded into each cartridge and the flow was re-established to flush the extract containing PSTs which was collected into a new tube. Two ml of deionized water was loaded into the cartridge to complete the PSTs elution (final extract volume 3 mL). Cleaned extracts were filtered through 0.22 µm PTFE syringe filters prior to analysis. Cleaned-up extracts were re-run without oxidation to check that all interferences had been removed in the clean-up step.

Cultures (18–32 mL) from *A. minutum* strains (*n* = 45) were filtered through 25 mm diameter glass fiber filters (Whatman). An aliquot (2 mL) of each culture was taken for cell counts using Sedgwick-Rafter chambers after Lugol’s iodine fixation and dilution with filtered seawater.

Each filter, containing the *A. minutum* cells, was placed in an Eppendorf tube. Then, 750 µL 0.05 M acetic acid was added and the tubes were frozen at −20 °C until further use. Just before analysis, the contents of each tube were sonicated for 1 min at 50 watts and centrifuged at 5200× *g* and 10 °C for 10 min. The supernatant was collected in a clean Eppendorf tube. The extraction was repeated with another 750 µL 0.05 M acetic acid. Both supernatants were combined (final volume 1500 µL) and then filtered through 0.22 µm PTFE syringe filters prior to HPLC analyses.

The characterization of PSTs in the dinoflagellate strains and marine fauna samples was carried out by the HPLC-PCOX-FLD method of [35], with some modifications [36]. The UPLC equipment, post-column reaction system and reagents, mobile phases, gradient conditions and FLD detector wavelengths employed were as described in [36]. The LC column was a Waters XBridge^®^ Shield RP, 4.6 × 150 mm, 3.5 µm. The Certified Reference PSTs standards used were purchased from the National Research Council Canada (NRC-CRMs).

In order to determine the PSTs concentration in the samples, the external standard calibration procedure was used. Total samples toxicity was expressed as µg STX dihydrochloride equivalents/kg meat. The EFSA [14] STXs toxicity equivalency factors (TEFs) were used to calculate the toxicity contribution of each toxin.

## Figures and Tables

**Table 1:** Sampling data, *A. minutum* strains isolated from seawater samples taken in the bloom area and their toxic profile.

Strain Code	Sampling Date	Sampling Point and Coordinates	Toxic Profile
SA2B	28/06/2018	1: Samil Beach 42°12′38.0″ N, 8°46′34.3″ W	GTX3, GTX4
SA2C	28/06/2018		GTX2, GTX3, GTX4
SA3B	28/06/2018		-
SA3C	28/06/2018		GTX4
SA5B	28/06/2018		-
SFA	15/07/2018		GTX3, GTX4
S1	28/06/2018		GTX3, GTX4
PC4B	16/07/2018	2: Vigo marina (dockyards) 42°13′32.2″ N, 8°44′57.2″ W	GTX1, GTX2, GTX3, GTX4
PC4D	16/07/2018		GTX3, GTX4
P1FA	18/07/2018	3: Vigo marina (Beiramar) 42°13′41.5″ N, 8°44′17.2″ W	GTX4
P1FA2	18/07/2018		GTX4
P13A	18/07/2018		GTX4
P15C	18/07/2018		-
P14A	18/07/2018		-
P12A	18/07/2018		GTX3, GTX4
P2FB1	18/07/2018		-
P21B	18/07/2018		-
P2FA	18/07/2018		GTX3, GTX4
P21A	18/07/2018		GTX1, GTX2, GTX3, GTX4
P3FB2	18/07/2018		GTX2, GTX3, GTX4
P31C	18/07/2018		GTX2, GTX3, GTX4
P32D	18/07/2018		GTX1, GTX2, GTX3, GTX4
P3FA	18/07/2018		GTX3, GTX4
P31D	18/07/2018		-
P36A	18/07/2018		GTX4
P36B	18/07/2018		-
P4FA	18/07/2018		GTX3, GTX4
PFB3	18/07/2018		-
PB7F	09/07/2018	4: Vigo marina (inshore pier) 42°14′13.9″ N, 8°43′55.0″ W	GTX4
PB6F	09/07/2018		GTX4
PB9	09/07/2018		-
ALB6	17/07/2018	5: Vigo marina (A Laxe) 42°14′25.1″ N, 8°43′43.2″ W	GTX2, GTX3, GTX4
ALB7	17/07/2018		GTX2, GTX3, GTX4
ALC7	17/07/2018		GTX3, GTX4
P1	09/07/2018	6: Vigo marina (Náutico) 42°14′28.5″ N, 8°43′21.7″ W	GTX3, GTX4
P4	09/07/2018		GTX2, GTX3, GTX4
NA2	12/07/2018		GTX3, GTX4
NA3	12/07/2018		GTX3, GTX4
NA4	12/07/2018		GTX3, GTX4
NA5	12/07/2018		GTX1, GTX2, GTX3, GTX4
NB3	12/07/2018		-
NB4	12/07/2018		GTX4
NB5	12/07/2018		GTX4
NC2	12/07/2018		GTX3, GTX4
FP2	09/07/2018	7: Vigo marina (As Avenidas) 42°14′31.1″ N, 8°43′14.4″ W	GTX4

**Table 2:** Results from fish and invertebrate samples in which PSTs were quantified after analyses by HPLC-FLD-PCOX. (a) Next to 6: Náutico, (b) next to 3: Beiramar.

Samples and Their Codes	Sampling Point and Date	Tissues Employed for Analysis	Total Toxicity µg eq STX·diHCl/kg	Toxic Profile	
Grey mullet-1 25 cm, 380 g *Liza ramada*	Vigo (a) 10/07/18	Muscle, digestive tract, gonads	132	GTX3	
Grey mullet-2 25 cm, 420 g *Liza ramada*	Vigo (a) 10/07/18	Muscle, digestive tract, gonads	21.7	GTX3	
Grey mullet-3 40 cm, 750 g *Liza ramada*	Vigo (b) 11/07/18	Digestive tract	151	GTX3	
Grey mullet-6 37 cm, 550 g *Liza ramada*	Vigo (a) 20/07/18	Digestive tract	67.8	GTX3	
Mackerel-2 28 cm, 200 g *Scomber scombrus*	Vigo (b) 10/07/18	Muscle	27.6	GTX2, GTX3	
Mackerel-2 28 cm, 200g *Scomber scombrus*	Vigo (b) 10/07/18	Digestive tract	292	GTX1, GTX2, GTX3, GTX4	
Mussels-1 65 g homogenate *Mytilus galloprovincialis*	Vigo (a) 20/07/18	Whole body	1877	GTX1, GTX2, GTX3, GTX4, STX	
Ascidians-1 15 g homogenate *Ciona intestinalis*	Vigo (a) 20/07/18	Whole body	363	GTX1, GTX2, GTX3, GTX4	
Scallops-1 37 g homogenate *Chlamys varia*	Vigo (a) 20/07/18	Whole body	3437	GTX1, GTX2, GTX3, GTX4, STX, NEO	
Scallops-2 33 g homogenate *Chlamys varia*	Vigo (a) 20/07/18	Whole body	2670	GTX1, GTX2, GTX3, GTX4, STX, NEO	
Oysters-1 24 g homogenate *Ostrea edulis*	Vigo (a) 20/07/18	Whole body	485	GTX1, GTX2, GTX3, GTX4	
Oysters-2 5 g homogenate *Ostrea edulis*	Vigo (a) 20/07/18	Whole body	805	GTX1, GTX2, GTX3, GTX4	
Squids-5 17 g homogenate *Loligo vulgaris*	Vigo (a) 18/07/18	Whole body	16.3	GTX3
Starfish-1 *Marthasterias glacialis* 18 cm	Vigo (a) 20/07/18	Ambulacral groove	38.4	GTX2, GTX3	
Starfish-2 *Marthasterias glacialis* 16 cm	Vigo (a) 20/07/18	Ambulacral groove	57.8	GTX2, GTX3	
